# Epidemiological transition of primary cutaneous melanoma in a public hospital in Brazil (1999–2019)^☆^

**DOI:** 10.1016/j.abd.2022.02.004

**Published:** 2022-11-24

**Authors:** César Augusto Zago Ferreira, Lais Soares Ker Marques, Hélio Amante Miot, Juliano Vilaverde Schmitt

**Affiliations:** Department of Dermatology and Radiotherapy, Faculty of Medicine, Universidade Estadual Paulista, Botucatu, SP, Brazil

*Dear Editor,*

Despite accounting for only about 1% of all skin cancers, melanoma accounts for 90% of mortality from cutaneous malignancies, and the treatment of the advanced forms inflicts a significant budgetary impact on the health system.^1^ In recent decades, there has been a consistent increase in its incidence worldwide; however, its specific mortality has remained stable or has slightly decreased in most historical series.^2^

In the 2020-2022 triennium, INCA (Brazilian National Cancer Institute) estimated the diagnosis of 8,400 melanomas in Brazil (4 cases/100,000 inhabitants). As Brazilian longitudinal epidemiological data are scarce, this study aimed to verify the transition in the epidemiology of primary cutaneous melanomas diagnosed in a public university service in the hinterland of Brazil over a 21-year period.

A retrospective and analytical study of patients diagnosed with *in situ* or invasive melanoma was conducted between January 1999 and December 2019, in the Pathology Laboratory of Hospital das Clínicas, Medical School, Botucatu (FMB-Unesp). Demographic and histopathological data of the patients were collected, related to the characteristics of the neoplasm. The subgroups were compared by logistic models (binary or ordinal), the effect size was estimated by the odds ratio with its 95% confidence interval (95%CI), and the significance level was defined at p < 0.05. The project was approved by the Research Ethics Committee of the institution.

During this period, 615 primary cutaneous melanomas were diagnosed in 590 patients, of which 300 (50.8%) were female, and 24 (4.1%) had more than one melanoma during the period. The mean age (standard deviation) at diagnosis was 61.3 (15.8) years, ranging from 12 to 92 years. The incidence of primary cutaneous melanoma showed an average annual growth of 4.0% (95%CI 2.0% to 5.7%) at the institution per year, in the last 21 years. There was no difference in the proportion of elderly individuals or regarding sex, as a function of age (p > 0.68).

Table 1 shows the main characteristics of melanomas and their association with sex and age group. There was a predominance of cases occurring in the thoracic and cephalic regions. Tumors in the limbs were more frequent in women, while cephalic tumors were more frequent in the elderly and in men. The superficial spreading histopathological subtype was the most common, in addition to being associated with the female sex and ages under 60. Nodular melanomas predominated in men. Patients under 60 also had higher levels of histopathological invasion (1–3 mm).

**Table 1:** Main characteristics regarding sex and age group of primary cutaneous melanomas diagnosed in a university hospital: 1999 to 2019 (n = 615).

	Total		Female		Male		OR	95%CI	p-value	< 60 years		≥ 60 years		OR	95%CI	p-value
**Topography**																
Thorax	202	33%	83	27%	119	39%	0.58	0.42‒0.82	0.002	107	43%	95	26%	2.10	1.50‒2.96	< 0.001
Head/neck	193	31%	85	28%	108	35%	0.70	0.50‒0.99	0.043	50	20%	143	39%	0.38	0.27‒0.56	< 0.001
Upper limb	80	13%	53	17%	27	9%	2.16	1.33‒3.50	0.002	34	14%	46	13%	1.08	0.67‒1.74	0.742
Acral	72	12%	38	12%	34	11%	1.13	0.69‒1.85	0.626	29	12%	43	12%	0.98	0.59‒1.61	0.922
Lower limb	66	11%	47	15%	19	6%	2.73	1.59‒4.69	<0.001	31	12%	35	10%	1.32	0.79‒2.21	0.281
Other/Not classified	2	0%	2	1%	0	0%	‒	‒	‒	0	0%	2	1%	‒	‒	‒
**Histopathological subtype**																
Superficial spreading	256	42%	147	48%	109	36%	1.66	1.20‒2.29	0.002	139	55%	117	32%	2.62	1.89‒3.64	< 0.001
Lentigo maligna	174	28%	78	25%	96	31%	0.75	0.52‒1.06	0.102	30	12%	144	40%	0.21	0.14‒0.31	< 0.001
Acral lentiginous	64	10%	34	11%	30	10%	1.15	0.68‒1.92	0.607	23	9%	41	11%	0.79	0.46‒1.36	0.402
Nodular	60	10%	22	7%	38	12%	0.54	0.32‒0.94	0.029	26	10%	34	9%	1.12	0.66‒1.92	0.676
Other/Not classified	61	10%	27	9%	34	11%	‒	‒	‒	33	13%	28	8%	‒	‒	‒
**Breslow thickness**																
*In situ*	209	34%	106	37%	103	37%	1.00	‒	0.644	71	28%	138	38%	1.00	–	0.008
≤ 1 mm	173	28%	93	32%	80	29%	1.13	0.76‒1.69		83	33%	90	25%	1.79	1.19‒2.71	
1‒3 mm	81	13%	46	16%	35	13%	1.28	0.76‒2.14		40	16%	41	11%	1.90	1.13‒3.19	
> 3 mm	102	17%	43	14%	59	21%	0.71	0.44‒1.14		35	14%	67	18%	1.02	0.62‒1.67	
Not classified	50	8%	20	7%	30	10%	‒	‒	‒	22	9%	28	8%	‒	‒	‒
**Clark level**																
I	209	34%	106	34%	103	34%	1.00	‒	0.734	71	28%	138	38%	1.00	‒	0.048
II	96	16%	53	17%	43	14%	1.20	0.74‒1.95		46	18%	50	14%	1.79	1.09‒2.93	
III	132	21%	62	20%	70	23%	0.86	0.56‒1.33		63	25%	69	19%	1.78	1.14‒2.78	
IV	100	16%	49	16%	51	17%	0.93	0.58‒1.50		38	15%	62	17%	1.19	0.73‒1.95	
V	19	3%	12	4%	7	2%	1.67	0.63‒4.40		6	2%	13	4%	0.90	0.33‒2.46	
Not classified	59	10%	26	8%	33	11%	‒	‒	‒	27	11%	32	9%	‒	‒	‒

OR, Odds Ratio.

When comparing melanomas grouped into three seven-year periods (Table 2), adjusted for sex and age using ordinal logistic regression, acral and thoracic tumors increased in frequency, as did superficial spreading and acral lentiginous histopathological subtypes. On the other hand, tumors located on the head and neck, as well as lentigo maligna, had their frequency decreased during the periods. There was no change in tumor proportions according to histopathological invasion levels, and about 35% of the diagnosed melanomas measured > 1 mm.

**Table 2:** Evolution comparison of proportions related to cutaneous melanoma diagnosed in a university hospital: 1999-2019 (n = 615).

	1999‒2005 (n = 140)		2006‒2012 (n = 256)		2013‒2019 (n = 219)		OR^a^	95%CI	p-value^a^
**Topography**									
Thorax	33	24%	88	34%	81	37%	1.48	1.05‒2.08	**0.027**
Head and neck	61	44%	78	30%	54	25%	0.54	0.38‒0.76	**0.001**
Upper limb	24	17%	31	12%	25	11%	0.73	0.45‒1.17	0.189
Acral	9	6%	31	12%	32	15%	1.69	1.03‒2.75	**0.037**
Lower limb	13	9%	27	11%	26	12%	1.23	0.73‒2.06	0.436
Other/Not classified	0	0%	1	0%	1	0%	‒	‒	‒
**Histopathological subtype**									
Superficial spreading	49	35%	103	40%	104	47%	1.49	1.07‒2.07	**0.018**
Lentigo maligna	51	36%	77	30%	46	21%	0.55	0.38‒0.79	**< 0.001**
Acral lentiginous	8	6%	27	11%	29	13%	1.73	1.04‒2.87	**0.035**
Nodular	11	8%	26	10%	23	11%	1.19	0.71‒2.00	0.512
Other/Not classified	21	15%	23	9%	17	8%	‒	‒	‒
**Breslow thickness**									
*In situ*	38	27%	107	42%	64	29%	1.00	‒	0.522
≤1 mm	44	31%	66	26%	63	29%	0.96	0.64‒1.44	
1‒3 mm	16	11%	28	11%	37	17%	1.42	0.84‒2.41	
>3 mm	25	18%	38	15%	39	18%	1.06	0.66‒1.70	
Not classified	17	12%	17	7%	16	7%			
**Clark level**									
I	38	27%	107	42%	64	29%	1.00	‒	0.960
II	24	17%	35	14%	37	17%	1.02	0.62‒1.69	
III	31	22%	48	19%	53	24%	1.11	0.71‒1.74	
IV	29	21%	35	14%	36	16%	0.89	0.55‒1.45	
V	4	3%	9	4%	6	3%	0.97	0.38‒2.47	
Not classified	14	10%	22	9%	23	11%	‒	‒	‒

OR, Odds Ratio.

a Results adjusted for sex and age.

Fig. 1 depicts the perceptual map, estimated by the multivariate technique of multiple correspondence analysis, which simultaneously adjusts for sex, age group, histopathological type, and Breslow thickness. The multivariate model consisting of two dimensions explained 62% of the total variation (32% and 29% inertia), allowing the identification of close relationships between variables and continuity between the categories. Melanomas of intermediate thickness (1–3 mm), acral, superficial spreading subtype, and age under 60, were closer to the most recent period (2013-2019) of follow-up. While the most remote period (1999-2005) was closer to the elderly, lentigo maligna and tumors < 1 mm. The nodular subtype was associated with higher levels of invasion, without approaching the time of follow-up, sex, or age group.

**Figure 1:**
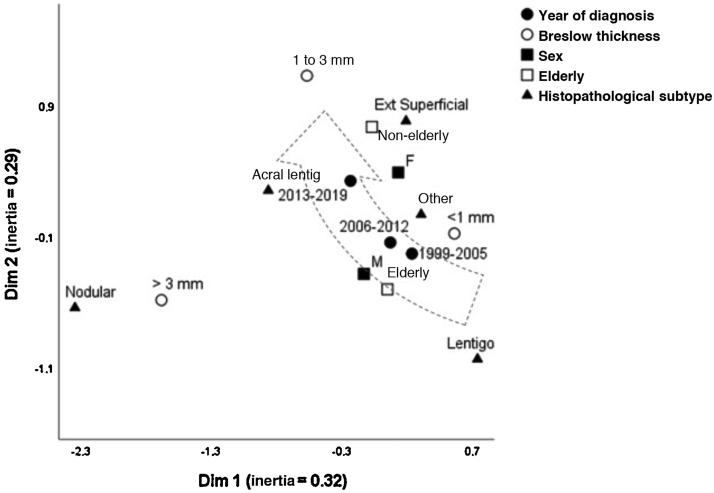
Perceptual map (multiple correspondence analysis) of cutaneous melanoma cases diagnosed at a university hospital: 1999–2019 (n = 615).

In this series, the increased percentage seen in melanomas diagnosed at the institution was greater than the population growth in the region, suggesting an increase in incidence. However, despite awareness campaigns, invasive melanomas still comprise an important fraction of the tumors diagnosed in the institution, and the results do not show a reversal of this scenario. In fact, there is multifactorial evidence for the transition in the epidemiology of melanomas in different international series, with justification ranging from overdiagnosis to photoexposure profiles, population aging, racial miscegenation, and prevention campaigns.^3^, ^4^

In this population, despite increasing aging, the reduction in the proportion of melanomas in the elderly, and in the lentiginous subtypes prevalent in the head/neck region, may reflect a better occupational photoprotection pattern, in addition to the urbanization process that Brazil has been experiencing since the 1970s.^5^ This melanoma profile is associated with slow growth during the horizontal phase, and less aggressive behavior.

On the other hand, the increase in acral forms and tumors in young adults may reflect the population miscegenation, since these forms are more common in adult African and Asian descendants. This melanoma profile has a more aggressive behavior, with an early vertical phase.^6^

The fraction of nodular melanomas in the institution remained stable during the period, which had already been identified in other countries. These forms have an early invasive behavior, contribute to most of the specific mortality, and are not sensitive to early diagnosis based on campaigns that take into account the aspects of pigmentation, symmetry, or changes in preexisting nevi.^7^

In Goiânia (Brazilian central region), an increase in the incidence of melanomas was also identified between 1988 and 2000, in both sexes, despite higher mortality among men.^8^ This was also demonstrated in a death registry survey in the state of São Paulo (Southeastern Brazil).^9^ Similarly, another national study identified regional differences for melanoma-specific mortality trends, suggesting ethnic and environmental factors – which vary greatly across the country – that interfere with melanoma epidemiology.^10^

Population screening campaigns are an important tool adopted worldwide^11^ and Brazil has promoted them annually for two decades. Additional strategies such as raising podiatrists awareness and body mapping, since multiple nevi increase the chance of superficial spreading melanomas, are also important for an early diagnosis.

This study has limitations due to its retrospective characteristic, the fidelity of medical records, and the lack of longitudinal data regarding the patients clinical outcome. Moreover, the institution's pathology laboratory did not process all the melanoma cases in the region, which did not allow inferences to be made regarding its incidence. Nevertheless, the hospital centralizes all the histopathological diagnoses of the Brazilian public health system (SUS, *Sistema Único de Saúde*), in this geographical area allowing a representative sample of this regional population.

In conclusion, primary cutaneous melanoma has shown changes in the epidemiological profile in the last 21 years, in this institution. Prevention campaigns should alert for acral subtypes, and those located on the thorax, especially in individuals under 60 years of age, aiming at attaining an early diagnosis, considering the characteristics that have become more prevalent.

## Financial support

None declared.

## Authors' contributions

César Augusto Zago Ferreira: Design and planning of the study; collection, analysis, and interpretation of data; drafting and editing of the manuscript; approval of the final version of the manuscript.

Lais Soares Ker Marques: Data collection; approval of the final version of the manuscript.

Hélio Amante Miot: Design and planning of the study; analysis and interpretation of data; review of the manuscript; approval of the final version of the manuscript.

Juliano Vilaverde Schmitt: Design and planning of the study; analysis and interpretation of data; review of the manuscript; approval of the final version of the manuscript.

## Conflicts of interest

None declared.
